# Supplementary material for: Very low prevalence of validated kelch13 mutations and absence of hrp2/3 double gene deletions in South African malaria-eliminating districts (2022–2024)
Source: medRxiv. 2025 Apr 1:2025.03.31.25324948. Preprint. [Version 1] doi: 10.1101/2025.03.31.25324948 (PMC11998825; doi:10.1101/2025.03.31.25324948)
Supplement: 1 [file NIHPP2025.03.31.25324948V1-supplement-1.pdf]

medRxiv preprint doi: https://doi.org/10.1101/2025.03.31.25324948; this version posted April 1, 2025. The copyright holder has placed this preprint (which was not certified by peer review) in the Public Domain. It is no longer restricted by copyright. Anyone can legally share, reuse, remix, or adapt this material for any purpose without crediting the original authors.

Supplementary Tables

Table S1: Malaria species identified by standard PCR and the MAD^{4}HatTeR protocols in KwaZulu-Natal and Mpumalanga provinces, during the 2022/2023 and 2023/2024 malaria seasons.

PARASITE SPECIES	PROVINCE						OVERALL		
	KWAZULU-NATAL (KZN)			MPUMALANGA (MPN)					
	2022/2023	2023/2024	p-value	2022/2023	2023/2024	p-value	KZN	MPN	p-value
**PCR**	N = 573	N = 569		N = 1,350	N = 1,979	0.4	N = 1,142	N = 3,329	>0.9
Pf[a]	573 (100%)	569 (100%)		1,349	1,979		1,142	3,328	
Pf + Po[b]	0 (0%)	0 (0%)		1 (<0.1%)	0 (0%)		0 (0%)	1 (<0.1%)	
**MAD4HatTTeR**	N = 132	N = 162	0.7	N = 302	N = 710	0.8	N = 294	N = 1,012	<0.001
Pf[a]	119 (90%)	148 (91%)		297 (98%)	691 (97%)		267 (91%)	988 (98%)	
Pf + Pm[c]	9 (6.8%)	8 (4.9%)		4 (1.3%)	12 (1.7%)		17 (5.8%)	16 (1.6%)	
Pf + Pm + Poc[d]	0 (0%)	2 (1.2%)		0 (0%)	0 (0%)		2 (0.7%)	0 (0%)	
Pf + Pm + Pow[e]	0 (0%)	1 (0.6%)		0 (0%)	0 (0%)		1 (0.3%)	0 (0%)	
Pf + Poc[f]	2 (1.5%)	2 (1.2%)		0 (0%)	4 (0.6%)		4 (1.4%)	4 (0.4%)	
Pf + Poc + Pow[g]	0 (0%)	0 (0%)		0 (0%)	1 (0.1%)		0 (0%)	1 (<0.1%)	
Pf + Pow[h]	2 (1.5%)	1 (0.6%)		1 (0.3%)	2 (0.3%)		3 (1.0%)	3 (0.3%)	

[a] *Pf: Plasmodium falciparum*

[b] *Pf +Po: Plasmodium falciparum + Plasmodium ovale*

[c] *Pf +Pm: Plasmodium falciparum + Plasmodium malariae*

[d] *Pf + Pm + Po: Plasmodium falciparum + Plasmodium malariae + Plasmodium ovale*

[e] *Pf + Pm + Pow: Plasmodium falciparum + Plasmodium malariae + Plasmodium ovale wallikeri*

[f] *Pf + Poc: Plasmodium falciparum + Plasmodium ovale curtisi*

[g] *Pf + Poc + Pow: Plasmodium falciparum + Plasmodium ovale curtisi + Plasmodium ovale wallikeri*

[h] *Pf + Pow: Plasmodium falciparum + Plasmodium ovale wallikeri*

medRxiv preprint doi: https://doi.org/10.1101/2025.03.31.25324948; this version posted April 1, 2025. The copyright holder has placed this preprint (which was not certified by peer review) in the Public Domain. It is no longer restricted by copyright. Anyone can legally share, reuse, remix, or adapt this material for any purpose without crediting the original authors.

Table S2: Cross-tabulation of socio-demographic characteristics between confirmed falciparum samples and samples successfully sequenced using MAD[4]HatTeR

Socio-demographic	Total qPCR N = 2,338 (%)	Successfully N = 1,791 (%)	p-value
**Province**			
KwaZulu-Natal	701 (30%)	537 (30%)	>0.9
Mpumalanga	1,630 (70%)	1,248 (70%)	
Unknown	7	6	
**Malaria Season**			
2022-2023	916 (39%)	649 (36%)	<0.001
2023-2024	1,415 (61%)	1,136 (64%)	
Unknown	7	6	
**Gender**			
Female	640 (40%)	512 (41%)	0.017
Male	975 (60%)	730 (59%)	
Unknown	723	549	
**Age group**			
0 - <5	202 (8.8%)	163 (9.3%)	0.4
5 - <10	113 (4.9%)	92 (5.2%)	
10 - <20	294 (13%)	225 (13%)	
20 - <30	698 (30%)	521 (30%)	
30 - <40	484 (21%)	364 (21%)	
≥40	508 (22%)	392 (22%)	
Unknown	39	34	
**Case classification**			
Imported	1,678 (88%)	1,325 (88%)	0.8
Local	210 (11%)	170 (11%)	
Locally imported	13 (0.7%)	11 (0.7%)	
Unknown	437	285	

Table S3: Allele frequency for all genotypes assessed in falciparum samples from KwaZulu-Natal and Mpumalanga provinces, South Africa, during the 2022/2023 and 2023/2024 malaria seasons

Gene	Amino acid	Allele	PROVINCE			
			KWAZULU-NATAL		MPUMALANGA	
			2022-2023	2023-2024	2022-2023	2023-2024
dhfr	16/51/59	AIC	0.000237 (3.47e-08 - 0.00249)	0.00182 (4.76e-08 - 0.00694)	0.00412 (0.000877 - 0.0103)	0.00478 (0.00205 - 0.00873)
dhfr	16/51/59	AIR	0.997 (0.989 - 1)	0.998 (0.993 - 1)	0.989 (0.981 - 0.995)	0.988 (0.982 - 0.993)
dhfr	16/51/59	ANR	0.00289 (4.79e-05 - 0.011)	0	0.00666 (0.00232 - 0.0135)	0.00707 (0.00376 - 0.0114)
dhps	431/436/437	IAA	0.00712 (0.000772 - 0.0199)	0.000107 (1.58e-12 - 0.00119)	0.00129 (4.44e-05 - 0.00465)	0.00349 (0.00127 - 0.00703)
dhps	431/436/437	IAG	1.18e-11 (1.03e-12 - 7.13e-11)	0	0	4.23e-05 (2.94e-08 - 0.000407)
dhps	431/436/437	IFA	0.00266 (6.51e-05 - 0.0102)	0.000134 (3.05e-08 - 0.00127)	6.27e-05 (1.4e-12 - 0.000711)	0
dhps	431/436/437	IFG	5.31e-12 (1.02e-12 - 3.23e-11)	0	0	6.04e-05 (3.46e-08 - 0.000557)
dhps	431/436/437	ISA	0.0496 (0.0315 - 0.0751)	0.0215 (0.0107 - 0.0347)	0.0316 (0.0207 - 0.0448)	0.0463 (0.0357 - 0.0563)
dhps	431/436/437	ISG	0.941 (0.914 - 0.961)	0.978 (0.965 - 0.989)	0.967 (0.953 - 0.978)	0.95 (0.939 - 0.961)
dhps	431/436/437	ICA	0	0.000144 (3.36e-08 - 0.00145)	0	0
dhps	540/581	EA	0.946 (0.917 - 0.969)	0.967 (0.948 - 0.981)	0.952 (0.934 - 0.967)	0.942 (0.93 - 0.953)
dhps	540/581	KA	0.0543 (0.0313 - 0.0829)	0.0331 (0.0186 - 0.0522)	0.0468 (0.0323 - 0.0632)	0.0558 (0.0454 - 0.068)
dhps	540/581	EG	0	0	0.0014 (3.63e-05 - 0.00505)	0.00182 (0.000359 - 0.00408)
dhps	540/581	ET	0	0	0	7.84e-05 (3.25e-08 - 0.000703)
dhps	613	A	1 (1 - 1)	0	0	0.999 (0.997 - 1)
dhps	613	S	4.69e-08 (3.32e-08 - 5.52e-08)	0	0	0.000659 (2.01e-05 - 0.00245)
dhps	613	V	0	0	0	0.00059 (1.84e-05 - 0.00242)
k13	494	I	0.00224 (4.73e-05 - 0.00762)	0.00935 (0.00284 - 0.0188)	0.00347 (0.000554 - 0.00901)	0.00115 (0.000159 - 0.00325)
k13	494	V	0.998 (0.992 - 1)	0.991 (0.981 - 0.997)	0.997 (0.991 - 0.999)	0.999 (0.997 - 1)
k13	578	A	0.992 (0.983 - 0.998)	0.986 (0.976 - 0.994)	0.985 (0.975 - 0.993)	0.995 (0.991 - 0.997)
k13	578	S	0.00756 (0.00218 - 0.0168)	0.0137 (0.00576 - 0.0245)	0.0144 (0.00696 - 0.0244)	0.00534 (0.00257 - 0.00929)
k13	578	T	7.37e-05 (1.04e-12 - 0.000923)	0	0.000128 (3.3e-08 - 0.00113)	6.11e-05 (3.22e-08 - 0.000534)
mdr1	182/184/186	GFW	0.432 (0.365 - 0.497)	0.479 (0.421 - 0.532)	0.494 (0.451 - 0.537)	0.48 (0.449 - 0.51)
mdr1	182/184/186	GYW	0.568 (0.503 - 0.635)	0.521 (0.468 - 0.579)	0.506 (0.463 - 0.549)	0.52 (0.49 - 0.551)
mdr1	86	F	0	0	8.08e-12 (1.02e-12 - 2.73e-11)	0
mdr1	86	N	0	0	0.995 (0.988 - 0.999)	1 (1 - 1)
mdr1	86	Y	0	0	0.00516 (0.00115 - 0.0122)	1.61e-09 (0 - 2.9e-08)
mdr2	492	I	0.812 (0.762 - 0.862)	0.832 (0.791 - 0.871)	0.832 (0.8 - 0.862)	0.811 (0.787 - 0.833)
mdr2	492	V	0.188 (0.138 - 0.238)	0.168 (0.129 - 0.209)	0.168 (0.138 - 0.2)	0.189 (0.167 - 0.213)

medRxiv preprint doi: https://doi.org/10.1101/2025.03.31.25324948; this version posted April 1, 2025. The copyright holder has placed this preprint (which was not certified by peer review) in the Public Domain. It is no longer restricted by copyright. Anyone can legally share, reuse, remix, or adapt this material for any purpose without crediting the original authors.

Table S4: Prevalence of all *kelch13* mutation detection in KwaZulu-Natal and Mpumalanga provinces, South Africa, during the 2022/2023 and 2023/2024 malaria seasons

kelch13 mutation	PROVINCE							
	KWAZULU-NATAL				MPUMALANGA			
	2022/2023		2023/2024		2022/2023		2023/2024	
	n/N	%	n/N	%	n/N	%	n/N	%
V494I	3/147	2.04	7/182	3.85	3/198	1.52	0/424	0.00
P553L	0/69	0.00	0/151	0.00	0/171	0.00	1/371	0.27
P574L	0/175	0.00	0/182	0.00	0/210	0.00	2/432	0.46
A578S	5/175	2.86	8/182	4.40	11/210	5.24	6/432	1.39
A578T	1/175	0.57	0/182	0.00	0/210	0.00	1/432	0.23
C580R	0/175	0.00	1/182	0.55	1/210	0.48	0/432	0.00
V581A	0/175	0.00	2/182	1.10	0/210	0.00	0/432	0.00
L598S	0/174	0.00	1/178	0.56	1/203	0.49	0/416	0.00
N599D	0/174	0.00	0/178	0.00	0/203	0.00	1/416	0.24
E605V	0/174	0.00	1/178	0.56	0/203	0.00	0/416	0.00
K607E	0/174	0.00	1/178	0.56	1/203	0.49	0/416	0.00
K607R	0/174	0.00	0/178	0.00	3/203	1.48	2/416	0.48

Table S5: Multivariate logistic regression results for relevant demographics and drug resistance markers of interest

	dhps K540			*mdr1* Y184F			*mdr2* I492V		
**Characteristic**	**n/N (%)**	**OR[a] (95% CI[b])**	**p-value**	**n/N (%)**	**OR[a] (95% CI[b])**	**p-value**	**n/N (%)**	**OR[a] (95% CI[b])**	**p-value**
**Gender**	**N=950**			**N=1004**			**N=885**		
Female	35/396 (8.83%)	Ref[c]		281/416 (67.54%)	Ref[c]		104/361 (28.80%)	Ref[c]	
Male	51/554 (9.20%)	1.09 (0.68 - 1.73)	0,7	406/588 (69.04%)	1.06 (0.80 - 1.39)	0,7	169/524 (32.25%)	1.19 (0.88 - 1.60)	0,3
**Age Group**									
0-4	10/105 (9.52%)	Ref[c]		79/111 (71.17%)	Ref[c]		23/96 (23.96%)	Ref[c]	
5-9	6/57 (10.52%)	0.68 (0.22-2.09)	0,5	49/64 (76.56%)	1.50 (0.73 - 3.09)	0,3	22/53 (41.51%)	2.19 (1.05 - 4.56)	**0,037**
10-19	22/140 (15.71%)	1.34 (0.59 - 3.04)	0,5	97/140 (69.28%)	1.01 (0.58 - 1.76)	>0.9	42/125 (33.60%)	1.58 (0.86 - 2.90)	0,14
20-29	22/278 (7.91%)	0.82 (0.37 - 1.82)	0,6	206/291 (70.79%)	0.98 (0.60 - 1.59)	>0.9	75/266 (28.20%)	1.20 (0.70 - 2.06)	0,5
30-39	19/191 (9.94%)	0.97 (0.43 - 2.21)	>0.9	130/197 (65.98%)	0.81 (0.49 - 1.35)	0,4	61/176 (34.66%)	1.69 (0.96 - 2.97)	0,069
40+	7/179 (3.91%)	0.32 (0.12 - 0.89)	**0,029**	126/201 (62.68%)	0.70 (0.42 - 1.16)	0,2	50/169 (29.59%)	1.33 (0.75 - 2.37)	0,3
**Case classification**									
Imported	61/834 (7.31%)	Ref[c]		616/883 (69.76%)	Ref[c]		238/779 (30.55%)	Ref[c]	
Local	24/110 (21.81%)	3.58 (2.04 - 6.30)	**<0.001**	68/115 (59.13%)	0.62 (0.40 - 0.94)	**0,023**	35/100 (35.00%)	1.06 (0.67 - 1.69)	0,8
Locally imported	1/6 (16.66%)	3.54 (0.38 - 32.6)	0,3	3/6 (50.00%)	0.50 (0.10 - 2.53)	0,4	0/6 (0.00%)	0.00 (0.00 - inf)	>0.9

[a] *OR: Odds Ratio*

[b] *95% CI: 95% Confidence Interval*

[c] *Ref: Reference group*

Table S6: Within host genetic diversity in samples in KwaZulu-Natal and Mpumalanga provinces, South Africa, during the 2022/2023 and 2023/2024 malaria seasons.

Variable	PROVINCE				CASE CLASSIFICATION	
	KWAZULU-NATAL		MPUMALANGA		Imported	LOCAL
	2022/2023	2023/2024	2022/2023	2023/2024	N= 1322	N = 170
COI[a]	3.37 (3.20-3.52)	2.92 (2.81-3.01)	2.76 (2.69-2.87)	2.86 (2.81-2.91)	2.89 (2.85-2.95)	2.31 (2.22-2.41)
eCOI[b]	2.11 (2.08-2.13)	1.97 (1.94-1.99)	2.03 (2.02-2.04)	2.03 (2.02-2.04)	2.04 (2.03-2.04)	1.75 (1.74-1.78)
% polyclonal	72.59% (69.38-75.19%)	70.22% (67.74-72.05%)	67.25% (65.47-70.33%)	69.05% (67.79-70.48%)	69.31 % (68.23-70.73%)	61.42% (58.82-64.12%)

[a]COI: complexity of infection

[b]eCOI: effective COI

Mean (95% confidence interval)

medRxiv preprint doi: https://doi.org/10.1101/2025.03.31.25324948; this version posted April 1, 2025. The copyright holder has placed this preprint (which was not certified by peer review) in the Public Domain. It is no longer restricted by copyright. Anyone can legally share, reuse, remix, or adapt this material for any purpose without crediting the original authors.

Table S7: Multivariate logistic regression results for relevant demographics and COI, eCOI and polyclonality

**Outcome**	**COI[a]**		**eCOI[b]**		**Probability polyclonal >0.05**	
**Coefficients**	**Estimate**	**p value**	**Estimate**	**p value**	**Estimate**	**p value**
**Intercept**	1,51E+00	**<0.001**	8,49E-01	**<0.001**	1,49E+00	**<0.001**
**KZN-2023/2024**	-5,80E-01	**<0.001**	-4,73E-01	**0,007**	-7,11E-01	0,083
**MPN-2022/2023**	-4,10E-01	**0,001**	-2,13E-01	0,197	-1,96E-01	0,623
**MPN-2023/2024**	-2,80E-01	**0,010**	-2,14E-01	0,151	-2,85E-01	0,446
**log10(parasitemia)**	-1,77E-01	**<0.001**	-1,39E-01	**<0.001**	-1,03E-07	0,409
**Sequencing_depth**	2,71E-08	0,100	4,19E-08	0,035	2,34E-08	0,558
**Age:5-9**	1,92E-01	0,186	1,49E-01	0,402	NA	NA
**Age:10-19**	-2,13E-02	0,869	-1,33E-01	0,405	NA	NA
**Age:20-29**	-4,25E-03	0,970	-1,23E-01	0,379	NA	NA
**Age:30-39**	-7,49E-02	0,530	-2,47E-01	0,096	NA	NA
**Age:40+**	-2,43E-01	**0,045**	-4,46E-01	**0,004**	NA	NA
**Local**	-3,45E-01	**<0.001**	-2,80E-01	**0,028**	-4,89E-01	**0,033**
**Locally imported**	-1,30E-01	0,671	-3,32E-01	0,459	3,48E-02	0,966

[a]COI: complexity of infection

[b]eCOI: effective COI.

medRxiv preprint doi: https://doi.org/10.1101/2025.03.31.25324948; this version posted April 1, 2025. The copyright holder has placed this preprint (which was not certified by peer review) in the Public Domain. It is no longer restricted by copyright. Anyone can legally share, reuse, remix, or adapt this material for any purpose without crediting the original authors.

Supplementary Figures

Figure S1:

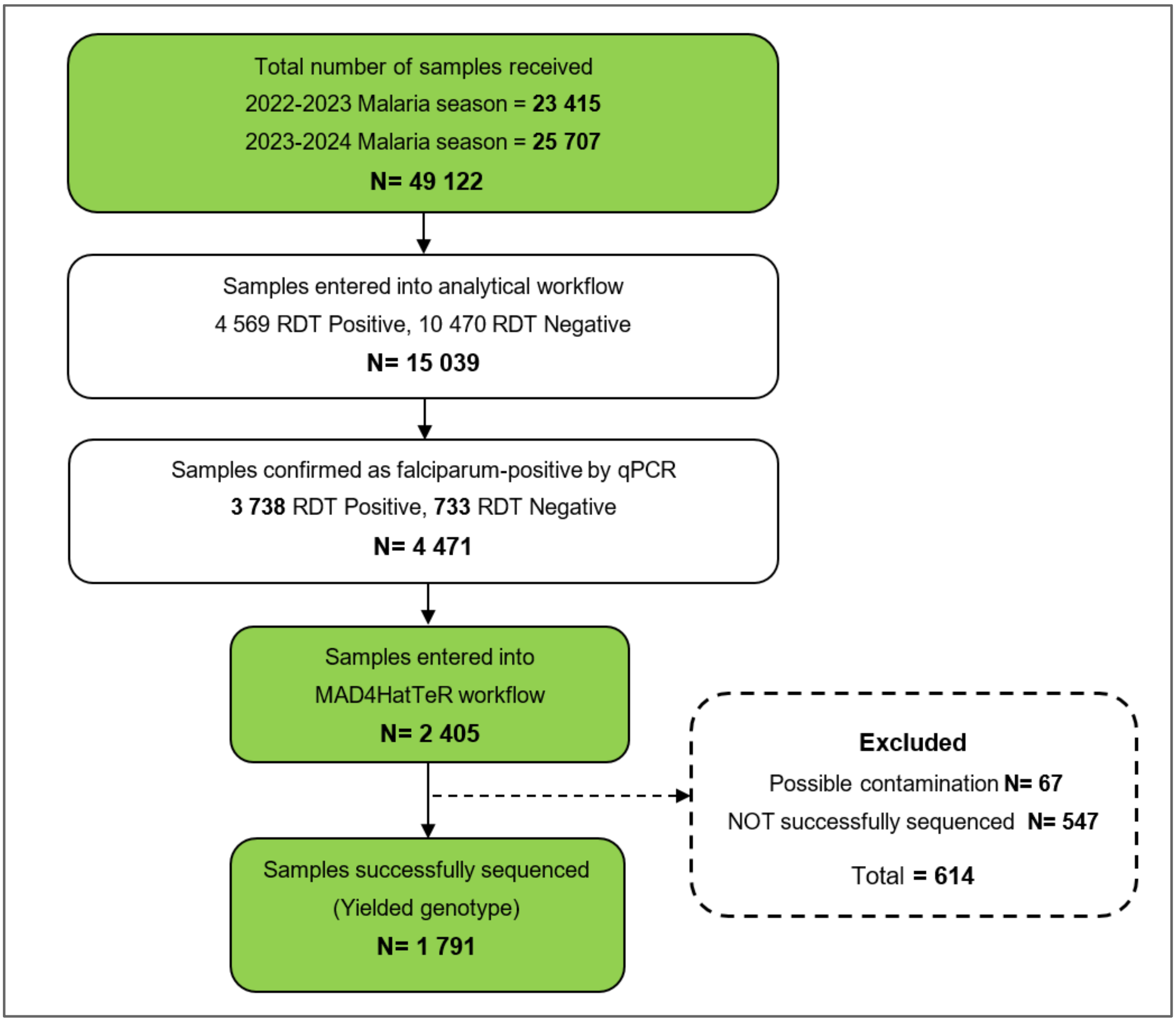

Figure S1: The number of samples received by the Central Laboratory during the 2022/2023 and 2023/2024 malaria seasons, the number entered into analytical workflow, and the number successfully sequenced through the MAD^{4}HatTer workflow.

medRxiv preprint doi: https://doi.org/10.1101/2025.03.31.25324948; this version posted April 1, 2025. The copyright holder has placed this preprint (which was not certified by peer review) in the Public Domain. It is no longer restricted by copyright. Anyone can legally share, reuse, remix, or adapt this material for any purpose without crediting the original authors.

Figure S2

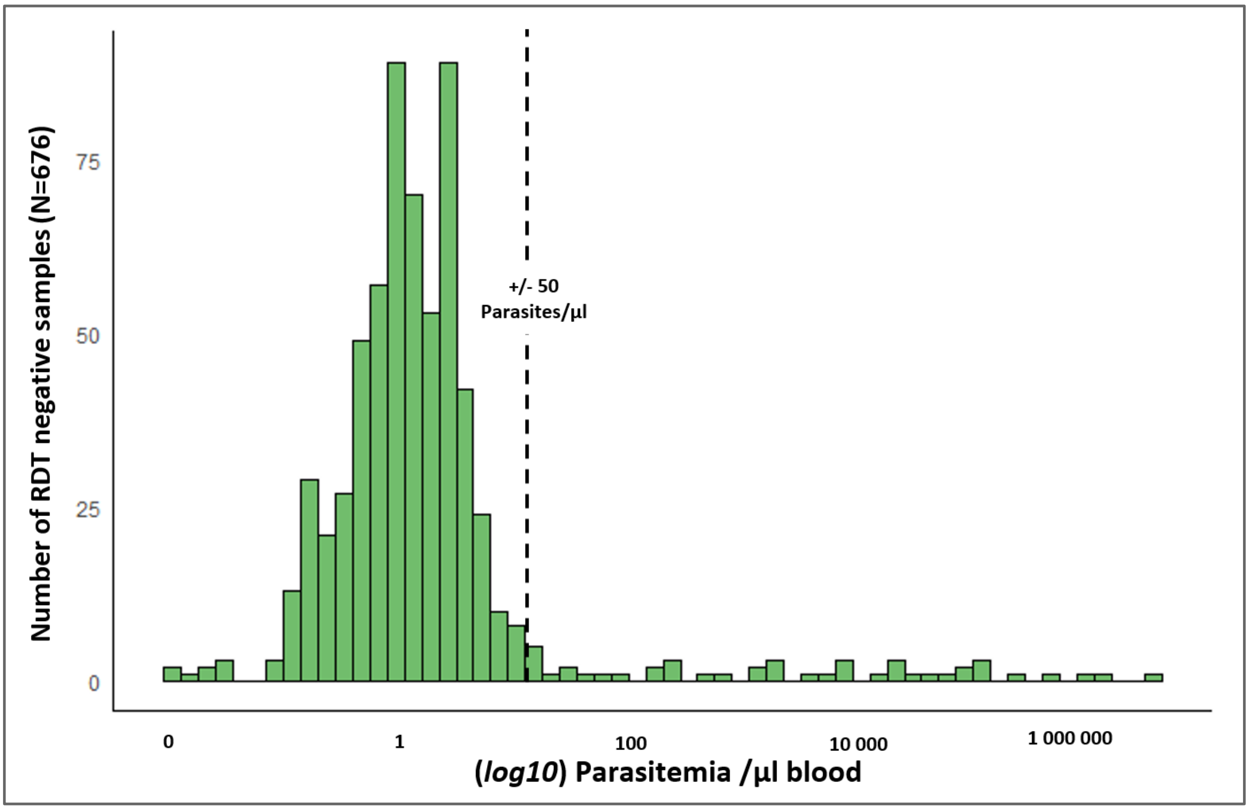

Figure S2: Parasitemia of RDT-negative but qPCR-positive samples detected over the study period

medRxiv preprint doi: https://doi.org/10.1101/2025.03.31.25324948; this version posted April 1, 2025. The copyright holder has placed this preprint (which was not certified by peer review) in the Public Domain. It is no longer restricted by copyright. Anyone can legally share, reuse, remix, or adapt this material for any purpose without crediting the original authors.

Figure S3

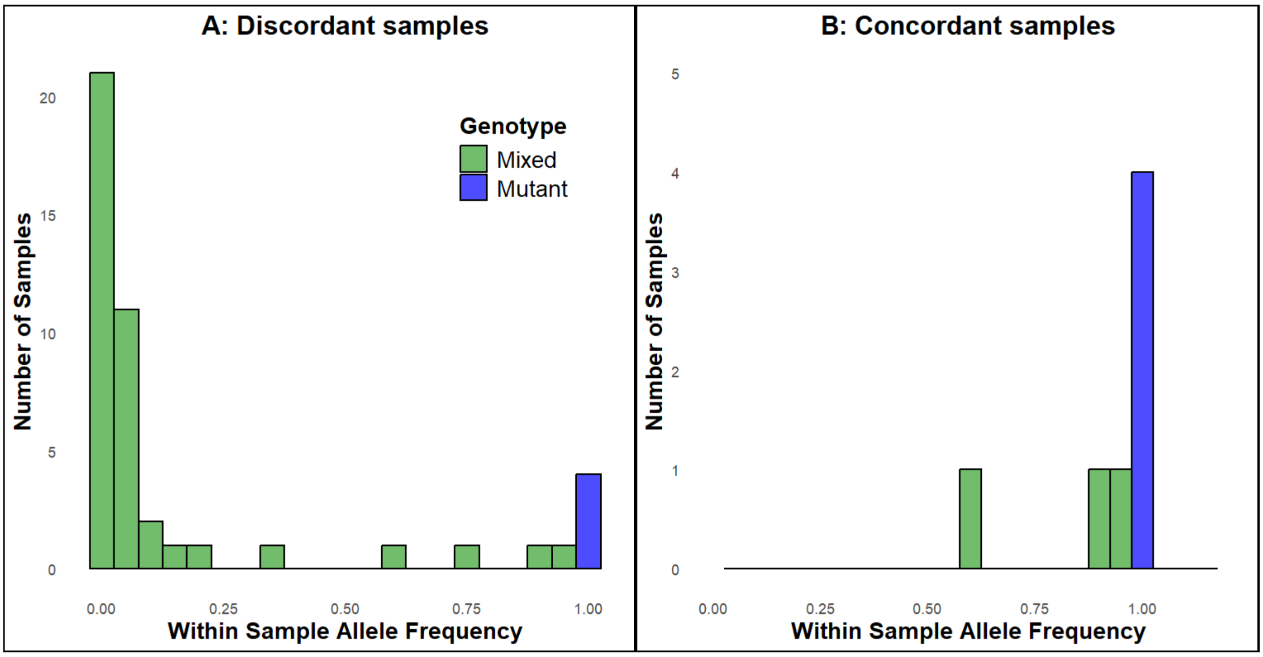

Figure S3: Within Sample Allele Frequencies (WSAF) for A) discordant and B) concordant samples containing a non-synonymous *kelch13* mutation
